# Immobilization of Laccase and Graphene Oxide in Langmuir–Blodgett Films as a Dual-Function Platform for Biosensors and Biosupercapacitors

**DOI:** 10.1021/acsomega.5c03493

**Published:** 2025-09-30

**Authors:** Felipe Merloto Marinho, Rebeca da Rocha Rodrigues, Kevin Figueiredo dos Santos, Laura Oliveira Péres, Danilo Alves Oliveira, José Roberto Siqueira, Luciano Caseli

**Affiliations:** † Laboratory of Hybrid Materials (LMH), Institute of Environmental, Chemical and Pharmaceutical Sciences, Federal University of São Paulo (UNIFESP), Diadema São Paulo 09913-030, Brazil; ‡ Laboratory of Applied Nanomaterials and Nanostructures (LANNA), Institute of Exact Sciences, Natural and Education, 74348 Federal University of Triângulo Mineiro (UFTM), Uberaba, Minas Gerais 38064-200, Brazil

## Abstract

The development of multifunctional hybrid films integrating biological and nanomaterial components offers promising avenues for advanced bioelectronic devices. In this study, we present Langmuir and Langmuir–Blodgett (LB) films composed of 1,2-dimyristoyl-*sn*-glycero-3-phosphate (DMPA), laccase (LAC), and graphene oxide (GO) as a versatile platform for both biosensing and energy storage applications. The films were thoroughly characterized using tensiometry, surface potential measurements, Brewster angle microscopy (BAM), and polarization-modulated infrared reflection–absorption spectroscopy (PM-IRRAS), revealing the successful incorporation of LAC and GO into DMPA monolayers without significant disruption of enzyme secondary structure. Enzymatic assays confirmed that LAC retained catalytic activity after immobilization, with GO enhancing both activity and diffusion dynamics. Electrochemical measurements demonstrated the films’ capacitive behavior, with GO significantly improving current density and charge retention in cyclic voltammetry and galvanostatic charge–discharge experiments. Additionally, the films exhibited excellent structural stability and electrochemical performance across multiple cycles. These findings demonstrate the potential of DMPA + LAC + GO LB films as a dual-function platform for phenolic compound biosensing and biosupercapacitor applications, highlighting the synergy between biocatalytic activity and electrochemical performance enabled by this hybrid architecture.

## Graphical abstract

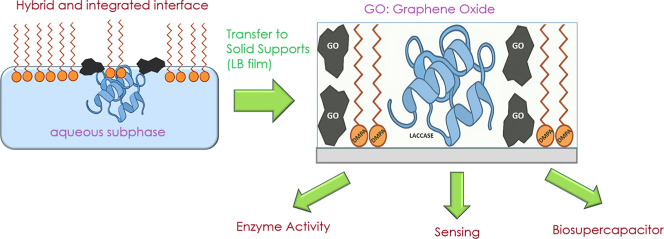

## 1 Introduction

The immobilization of enzymes within organized molecular films holds potential for advancing bioelectronic devices, particularly in biosensing, catalysis, and energy storage applications. In this regard, Langmuir–Blodgett (LB) films have proven to be an effective platform for assembling enzyme-functionalized lipid films with finely tunable properties.^1,2^ Graphene oxide (GO), with its excellent electrical conductivity and large surface area, is an attractive material for incorporation into these films, enhancing both their stability and functional performance. Additionally, biosupercapacitors based on these hybrid nanostructured films offer a promising path for bioelectronic applications, as they combine efficient charge storage with enzymatic activity, enabling the development of sustainable energy storage devices.^3–6^

Laccase, a multicopper oxidase enzyme characterized by the presence of four copper ions arranged in three distinct that work together in the electron transfer process during the reduction of molecular oxygen to water. This intrinsic redox property enables laccase to efficiently accept and donate electrons. In electrochemical devices, this activity translates into the ability to store and release electrical charge, especially when laccase is immobilized on conductive electrodes. As laccase is known for its ability to catalyze the oxidation of a wide range of phenolic compounds, has demonstrated potential in the development of biosensors.^7–12^ The combination of laccase and GO within lipid LB films offers a novel platform for detecting phenolic compounds, which are crucial in environmental and industrial settings. Notably, polyphenolic compounds play an essential role in determining the taste, flavor, and antioxidant properties of foods and beverages such as wine. Also, detection of phenolic compounds is important for both sustainability and pollution control because phenols are widely used in industrial processes—such as in the production of plastics, dyes, pharmaceuticals, and pesticides—and are common environmental pollutants. They are toxic to aquatic life and can accumulate in ecosystems, posing risks to human health and biodiversity. Monitoring phenols in water and food systems is essential for preventing environmental contamination, ensuring regulatory compliance, and promoting cleaner industrial practices. Additionally, phenol detection aligns with sustainable development goals by supporting safer production methods and protecting natural resources. While various sensors have been developed to measure these characteristics, there is a growing demand for more cost-effective, rapid, and accurate detection methods. The accurate quantification of polyphenols is critical for assessing product quality, authenticity, and nutritional value.

Beyond biosensing applications, laccase-based LB films may also hold promise for the development of biosupercapacitors.^13–15^ The rationale expected is when incorporated into biosupercapacitor systems, the enzyme contributes to pseudocapacitance processes, in which charge storage occurs via reversible surface redox reactions. Which means, laccase functions as a natural electroactive material, promoting charge accumulation, and its enzymatic activity combined with the high surface area and conductivity of GO, can contribute to efficient charge storage mechanisms, enhancing the performance of bioelectronic energy storage devices. By facilitating electron transfer and enabling stable enzyme immobilization, these hybrid films offer a sustainable approach to integrating biological components into energy storage systems, paving the way for innovative biosupercapacitor technologies.^3–6^

To advance this field, significant efforts have been made to immobilize laccase in thin films, a method essential for preserving the enzyme’s catalytic functionality. LB films, in particular, provide a highly organized and biomimetic platform that not only immobilizes enzymes but also enhances their stability and activity.^16–19^ This study aims to contribute to this growing field by presenting a novel method for stabilizing laccase from *Trametes versicolor* in Langmuir and LB films of 1,2-dimyristoyl-*sn*-glycero-3-phosphate (sodium salt) (DMPA). DMPA was selected due to its well-established ability to form stable Langmuir monolayers, offering an ideal matrix for enzyme immobilization. Its amphiphilic nature helps preserve the biological properties of the immobilized enzyme, while its ease of transfer to solid supports as LB multilayers further supports its use in bioelectronic applications.

We introduced graphene oxide into these films to explore its role in enhancing the bioelectronic properties of the devices, as well as the catalytic activity of the films. This proof-of-concept study investigates the feasibility of such hybrid films for bioelectronic applications as biosensors and biosupercapacitors. The surface chemistry of the floating monolayers, a critical factor in understanding the physicochemical properties of the supramolecular structures, was thoroughly examined. This included studies of their thermodynamic properties (using tensiometry), rheological behavior, morphology, and molecular organization using vibrational spectroscopy. These insights are essential for assessing the potential bioelectronic and catalytic properties of the lipid-enzyme-GO films.

Consequently, this study represents a significant extension of our ongoing exploration into enzyme immobilization within Langmuir films, incorporating graphene oxide as an additive to monolayers composed of DMPA and LAC. Our approach involved a comprehensive investigation of the surface chemistry of floating monolayers, assessing their transfer to solid supports using the LB methodology. This research represents a study on a Langmuir and Langmuir–Blodgett system that specifically investigates the impact of graphene oxide (GO) and laccase (LAC) on the physicochemical properties of Langmuir monolayers. Additionally, it explores the potential application of LB films as active hybrid nanofilms for phenolic compound biosensors and biosupercapacitors for energy storage.

## 2 Materials and Methods

Laccase from *Aspergillus* sp*.* and graphene oxide (GO) were obtained commercially from Sigma-Aldrich. Laccase was dispersed in 0.1 M phosphate buffer (pH 7.4) to a concentration of 1.0 mg/mL and GO in water to prepare a suspension of 4.4 mg/mL. DMPA (1,2-dimyristoyl-*sn*-glycero-3-phosphate) (Figure 1) was acquired from avanti polar lipids and dissolved in chloroform (CHCl_3_) at 0.5 mg/mL. Water was purified using a Milli-Q system from Millipore, achieving a resistivity of 18.2 MΩ·cm, and chloroform was obtained from LabSynth. The temperature was maintained at 25 ± 1 °C for all experiments.

**Figure 1:**
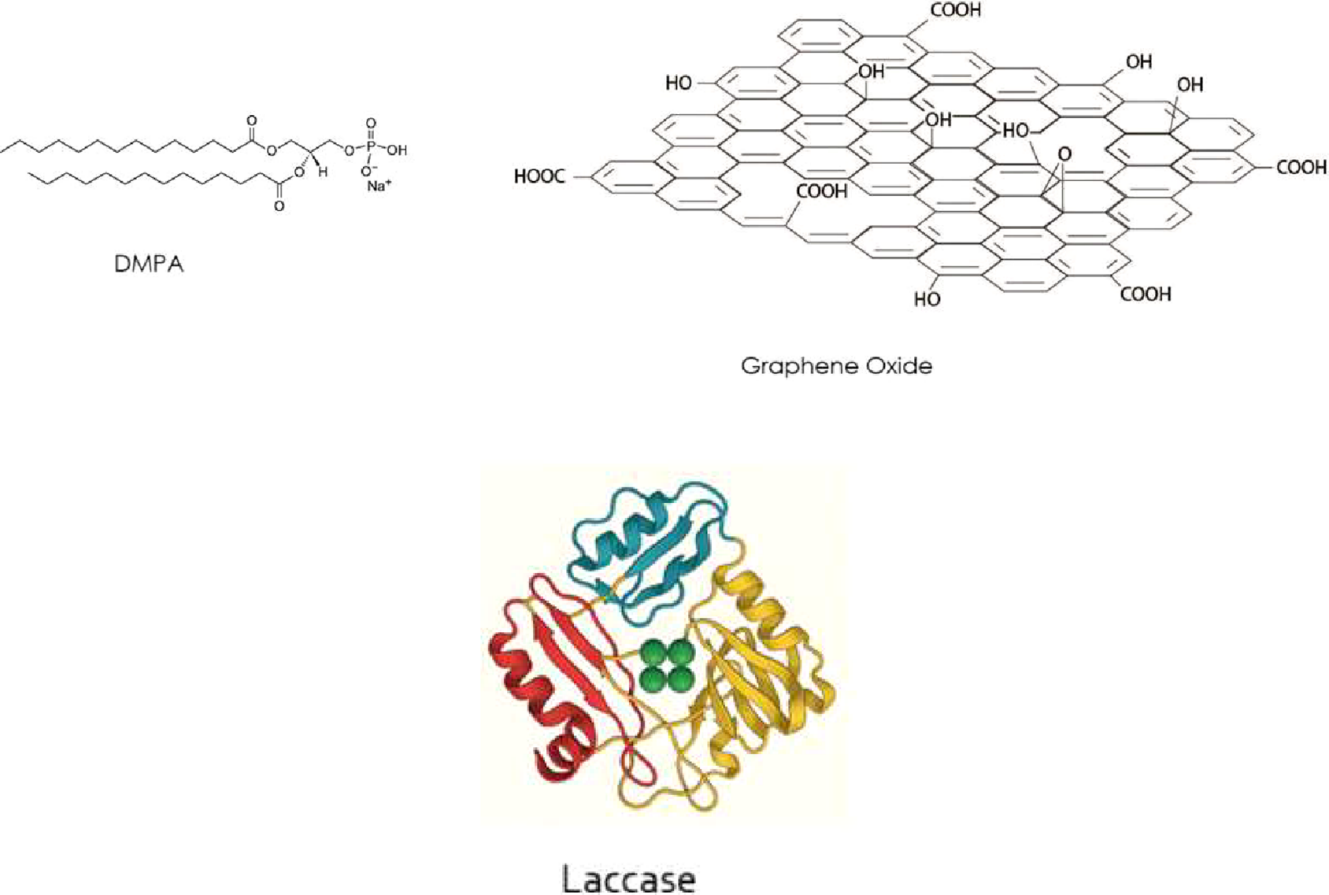
Molecular structures of DMPA, graphene oxide, from Laccase from *Aspergillus* sp. Based on.^20^ The structures were reconstructed from well-established reference models.

Langmuir monolayers and Langmuir–Blodgett (LB) films were prepared using a Langmuir trough (KSV-model Mini) containing 220 mL of water. DMPA solutions were spread at the air–water interface, followed by a 10 min waiting period to allow solvent evaporation. For experiments involving GO, the aqueous subphase was prepared with a GO suspension inserting 100 μL of the stock solution and letting it homogenize. In cases involving laccase, variable aliquots of the enzyme stock solution were introduced into the aqueous subphase, and a 10 min diffusion period was allowed for equilibration. The first characterization (surface pressure–area isotherms as described below) permitted the choice of the best proportion of enzyme inserted for further experiment, which was 4 μL of the stock solution.

Monolayer characterization included surface pressure measurements, surface potential analysis, polarization-modulated infrared reflection–absorption spectroscopy (PM-IRRAS), and Brewster angle microscopy (BAM). Spectrophotometry and microscopy were performed using a PMI 550 instrument and a mini-BAM, respectively, both from KSV Instruments. To control the surface density, two movable barriers symmetrically compressed the monolayers at a rate of 10 cm/min (5 Å^2^ molecule^–1^·s^–1^). Surface pressure was continuously monitored using a Wilhelmy plate made of filter paper, while surface potential was measured using a Kelvin probe.

Compression–decompression isotherms (π–A) were generated by compressing the monolayers up to 30 mN/m, just below the collapse point, and then expanding them back to the maximum trough area. Two compression–expansion cycles were performed. For stability assessments, the monolayers were compressed to 30 mN/m, and surface pressure was monitored over time under constant area conditions.

Dilatational rheology experiments were conducted by compressing the monolayers to 30 mN/m, followed by a 10 min stabilization period. Then, the monolayers were subjected to 10 compression–expansion cycles with a 1% area variation at a frequency of 20 mHz. The complex surface dilatational modulus (E*) was calculated using the formula E* = -A­(Δπ/Δ*A*), averaged over the cycles. The phase shift (θ) between surface pressure (stress) and area (strain) was determined, yielding the elastic (storage) modulus (*E*′ = E* sinθ) and viscous (loss) modulus (*E*″ = E* cosθ).

PM-IRRAS spectra were collected from monolayers compressed to 30 mN/m and stabilized for 10 min, with oscillation of the barriers. Simultaneously, BAM images were captured during compression up to 20 mN/m, with representative images recorded over a minimum of 1 h. A minimum of 6000 scans were performed at an incident angle of 80° to the surface normal, and all spectra were baseline-corrected by subtracting the reference spectrum (air–water interface before lipid spreading).

Langmuir–Blodgett films were deposited by transferring monolayers from the air–water interface onto precleaned solid glass supports at a surface pressure of 30 mN/m. The supports were cleaned with KOH in ethanol for 5 min before being introduced into the aqueous subphase. Following monolayer formation and compression to 30 mN/m, the supports were vertically withdrawn at a rate of 5 mm/min. For multilayer deposition, the supports were allowed to dry for 10 min after each deposition cycle before being immersed back into the subphase for additional layer transfers at the same speed. The number of support passages determined the number of deposited layers, and transfer ratios in the range of 1.0 ± 0.5 indicated the efficiency of deposition.

Subsequent characterization of the LB films involved PM-IRRAS at an incident angle of 80° with at least 12,000 scans, as well as quartz crystal microbalance (QCM) analysis (SRS- Stanford Research System, model QCM 200). The enzyme’s catalytic activity was measured following a previously established method,^21^ using UV–visible spectroscopy (Hitachi, model U2001). Syringaldazine (98%, Sigma-Aldrich) was used as the enzymatic substrate, following established protocols from the literature.^21^ Syringaldazine was dissolved in 0.05 M DMF (Synth), and the experiments were conducted at 30 °C. An initial baseline spectrum was recorded prior to film introduction. Subsequently, spectra were collected at predetermined time intervals after the film was immersed in the syringaldazine solution to monitor the increase in the absorption band corresponding to the oxidation product. The progression of the absorbance at the wavelength associated with the syringaldazine oxidation product was tracked over time. From these data, a graph of absorbance versus time was constructed, and the linear portion of the graph was used to determine the enzymatic activity. The slope of the linear region, corresponding to the rate of increase in absorbance, was calculated and used as the numerical value representing the enzymatic activity of the respective film. Atomic force microscopy (AFM) imaging was performed using a Nanoscope IIIA system in tapping mode. Mica substrates were used for film deposition, and images were acquired at a resonance frequency of approximately 300 kHz, with a scan rate of 1.0 Hz over areas of 5.0 × 5.0 μm^2^.

Cyclic voltammetry (CV) measurements were performed at room temperature using an AutoLab 128 N potentiostat and galvanostat (Metrohm Autolab, Switzerland). The experiments were conducted in a three-electrode electrochemical cell containing 0.1 M KCl as the supporting electrolyte. The LB films, deposited on an ITO substrate with an active geometric area of 0.5 cm^2^, functioned as the working electrode. A platinum foil (1.0 cm^2^) was used as the counter-electrode, while an Ag/AgCl (3 M KCl) electrode served as the reference electrode. To ensure proper deoxygenation, the electrolyte solution was purged with N_2_ before each measurement.

The graphene oxide (GO) immobilized into the LB film was electrochemically reduced to rGO through 10 CV cycles at a scan rate of 50 mV/s, within a potential window ranging from −1.7 to 0.0 V (vs Ag/AgCl). The areal capacitances (Ca) were calculated from both CV and galvanostatic charge–discharge (GCD) measurements using eq 1.

1 $$Ca=\frac{1}{S\nu \Delta V}\int i\left(V\right)dV$$

where ∫*i*(*V*) d*V* represents the integral of the voltammogram, *S* is the geometric area (cm^2^) of the working electrode covered by the film, ν corresponds to the scan rate (V/s), and Δ*V* (*V*) is the potential window.

## 3 Results and Discussion

### 3.1 Floating Monolayers

Figure 2 presents the compression isotherms for DMPA, along with the effects of the sequential adsorption of laccase and graphene oxide (GO) from the aqueous subphase. First, it is important to note that neither laccase nor GO forms stable Langmuir monolayers on their own without the presence of DMPA. Both laccase and GO tend to partition between the air–water interface and the subphase, resulting in negligible increases in surface pressure upon compression, which indicates low surface activity. This observation justifies the need for a lipid monolayer, such as DMPA, to act as a matrix for enzyme and GO immobilization, facilitating the cospreading of these components.

**Figure 2:**
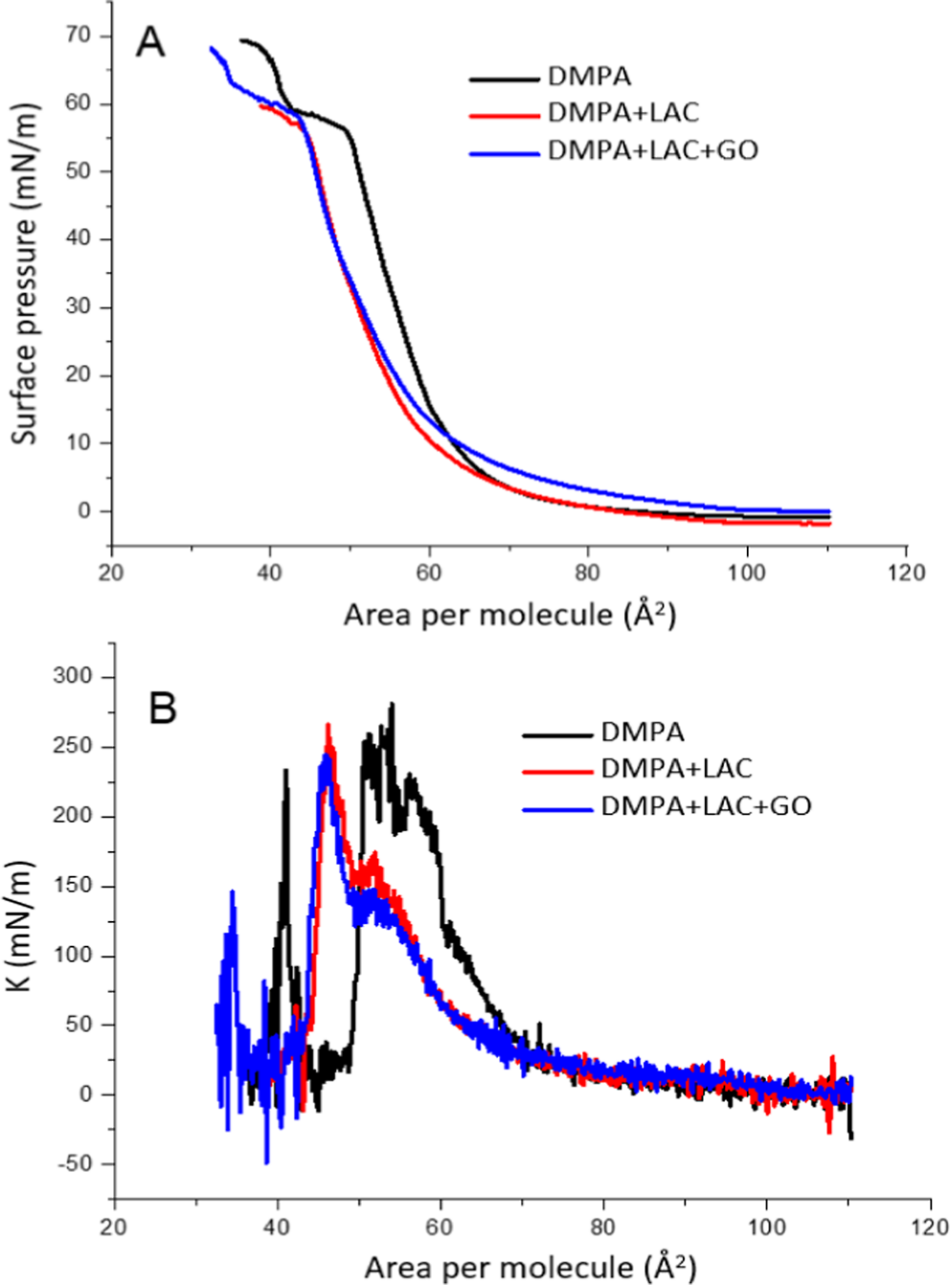
Compression isotherms for DMPA monolayers without or with laccase (0.18 μg/mL) and GO (2 μg/mL). (A) Surface pressure–area isotherms; (B): surface compression modulus–area isotherms. Subphase: 0.1 mol/L of KCl.

We also attempted to prepare monolayers using pure water in the subphase, but the resulting Langmuir–Blodgett (LB) films did not exhibit any detectable enzymatic activity, suggesting poor adsorption or inefficient transfer of laccase to the LB film. To address this, we explored using KCl in the subphase, as increased ionic strength is reported to enhance enzyme adsorption at the air–water interface without compromising catalytic activity.^22^ This approach yielded measurable enzyme activity, and thus, all results presented were obtained under these conditions. The concentration of laccase was optimized to ensure detectable changes in the tensiometric measurements, as well as reasonable levels of enzymatic and electrochemical activity. For consistency and comparison, the concentration of graphene oxide was chosen based on values reported in the literature.^15^

The surface pressure–area (π–A) isotherm of pure DMPA obtained under our experimental conditions is consistent with literature reports for monolayers formed at the air–water interface at similar subphase compositions and temperatures.^23,24^ It exhibits a characteristic liquid-expanded (LE) to liquid-condensed (LC) phase transition, identified by a pseudoplateau around 5 mN/m. This plateau reflects the onset of molecular reorganization and tighter packing as the film is compressed. Beyond this region, the isotherm shows a steady increase in surface pressure, indicative of the transition to a more ordered, condensed phase, with the monolayer collapsing at approximately 60 mN/m.

When laccase is incorporated into the subphase, the isotherm shifts toward lower mean molecular areas, suggesting increased molecular condensation within the monolayer. This behavior implies stronger lateral interactions between the DMPA molecules in the presence of laccase, which may be attributed to the enzyme acting as a bridging entity between adjacent DMPA headgroups. Although laccase has a net negative charge at the experimental pH (pI ≈4.0),^25^ it contains a heterogeneous distribution of charged and polar residues on its surface. These functional groups, along with the presence of counterions in the aqueous subphase, can reduce electrostatic repulsion among the negatively charged phosphate headgroups of DMPA, thereby promoting closer molecular packing and enhanced monolayer stability.

In contrast, the isotherm obtained for DMPA in the presence of graphene oxide (GO) and laccase shows a shift toward lower molecular areas, particularly in the final regions of compression. A slight expansion is observed in the beginning of the compression. This expansion indicates the successful incorporation of GO nanosheets within the monolayer, likely through physical entrapment or intercalation among the DMPA molecules. The amphiphilic nature and surface functionalization of GO (rich in oxygenated groups such as hydroxyls and carboxyls) can increase the spacing between DMPA molecules at low surface pressures due to steric and electrostatic interactions. However, as compression progresses and surface pressure increases, the isotherms of the DMPA/LAC and DMPA/LAC/GO systems begin to converge. Although the initial regions of the isotherms (at low surface pressures) for the DMPA/LAC and DMPA/LAC/GO systems are distinct—due to differences in molecular packing and film expansion—their behaviors become more similar as compression proceeds and surface pressure increases. Specifically, the gap in mean molecular area between the two isotherms narrows, and their slopes become comparable at higher pressures.

This convergence suggests a reorganization of the film components, with GO becoming more aligned and incorporated into the monolayer structure, allowing for a more compact and ordered packing regime at high compression states.

Together, these results demonstrate that both laccase and graphene oxide interact with DMPA at the molecular level, but in distinct ways: laccase promotes condensation through electrostatic screening and polar interactions, while GO initially disrupts molecular packing due to its bulk and functionalization, before becoming integrated into the compressed film architecture. The surface pressure (π-A) isotherms can be treated using the equation proposed by Davies and Rideal ${-A\left(\frac{\partial \pi }{\partial A}\right)}_{T}$ to obtain the surface compression modulus (*K*), as shown in Figure 1B. These values are usually associated with 2D states of the monolayer, and higher value indicates higher compressional elasticity of the film. Pure DMPA present *K* values as high as 250 mN/m indicating the liquid-condensed state of the film.^26^ Introducing laccase and then GO the maximum values does not change, only observing the left-shift as already noted in the π-A isotherms.

Figure 3 shows the compression–expansion isotherms for DMPA monolayers over two cycles. For pure DMPA on a saline subphase, we observe molecular accommodation after the first compression–expansion cycle, likely due to the incorporation of counterions into a more favorable geometry, which helps reduce lateral repulsions between molecules. In the presence of laccase (with or without GO), this molecular accommodation is less pronounced, as the enzyme already stabilizes the monolayer, as discussed in Figure 1. However, we observe greater hysteresis between the compression and expansion curves, likely due to increased viscous effects, which is consistent with previous reports in the literature.^27^

**Figure 3:**
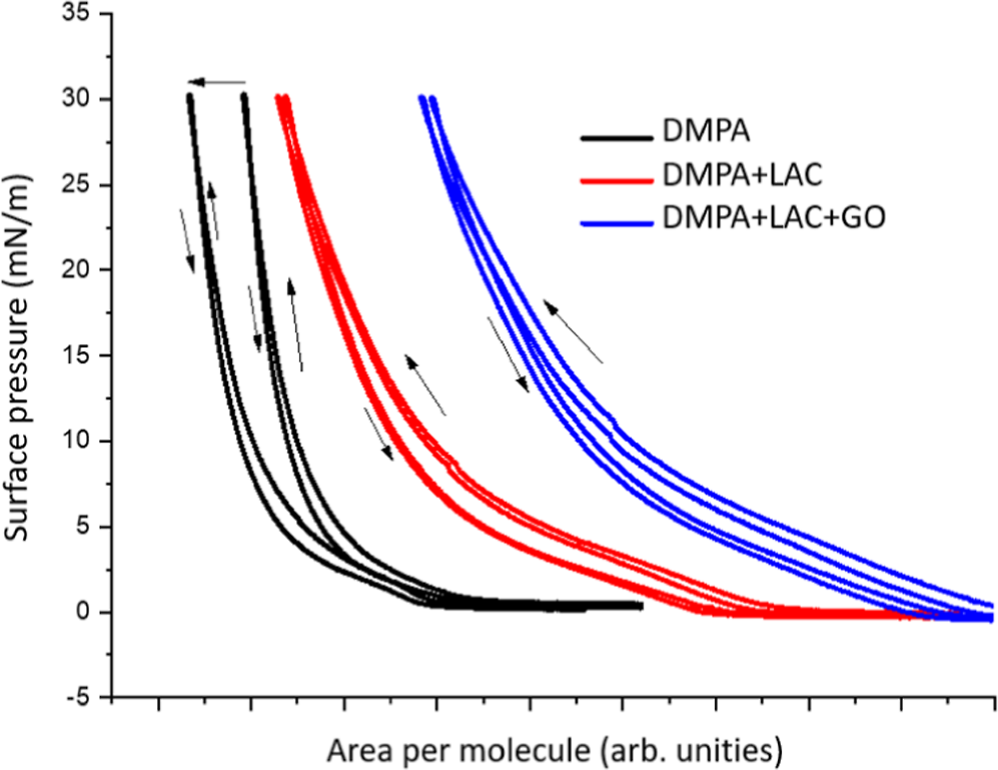
Compression–expansion surface pressure–area isotherms for DMPA monolayers without or with laccase (0.18 μg/mL) and GO (2 μg/mL).

Table 1 presents the dilatational data for the monolayers initially compressed to 30 mN/m, then allowed to relax for 10 min while the barriers moved back and forth, and subsequently subjected to short cycles of compression. Although the calculation of the complex dilatational modulus (E*) is mathematically identical to surface compressional elasticity (K), E* can yield different values. This difference arises because E* accounts for molecular relaxation phenomena occurring not only before the experiment but also during the short compression/expansion cycles.

**Table 1:** Interfacial Dilatational Viscoelastic Properties for DMPA Monolayers without or with Laccase (0.18 μg/mL) and GO (2μg/mL)^a^

	E* (mN/m)	*E*′ (mN/m)	*E*″ (mN/m)	θ (rad)
DMPA	134.10	125.36	47.63	0.36
DMPA + Laccase	111.91	105.43	37.54	0.34
DMPA + Laccase + GO	103.88	97.19	36.69	0.36

a Initial surface pressure = 30 mN/M, frequency = 20 mHz, and 1% of area variation.

The values presented in Table 1 were derived from oscillatory barrier modulation experiments using a Langmuir trough equipped with a Wilhelmy plate. After compressing the monolayers to the target surface pressure (typically 20 mN/m), the films were allowed to relax for 15 min to reach equilibrium. Subsequently, a sinusoidal area perturbation was applied with an amplitude of 1% of the initial area and a frequency of 2 mHz. The resulting surface pressure response was recorded and analyzed to determine the dilatational viscoelastic modulus E*.

This modulus reflects the film’s resistance to deformation under dynamic compression and expansion, and was calculated from the ratio of the amplitude of surface pressure variation to the amplitude of relative area change, according to the following equation

$$E^{*}=A\frac{\Delta \pi }{\Delta A}=E’+iE’’$$

where Δπ is the surface pressure variation, and *A*. Δ*A* is the relative area variation. The values represent the apparent dynamic elasticity of the monolayer, which encompasses both elastic and viscous contributions, though no phase angle decomposition was performed in this study.

The calculation of E* also incorporates the time lag between the stimulus (area variation) and the system’s response (surface pressure), resulting in a complex number represented by the storage modulus (*E*′) and loss modulus (*E*″), along with the phase angle (θ). Higher θ values indicate a greater contribution from the viscous component. For pure DMPA, a significant contribution from the viscous component is observed, with a phase angle of 0.36 rad, which is higher than values reported in the literature for pure lipids.^28^ This increased viscous contribution can be attributed to frictional forces between adjacent molecules in lateral motion, causing molecular interactions as they collide.

When laccase is added, the absolute value of E* decreases, indicating fluidization of the monolayer—a phenomenon not observed with uniaxial compression (Figure 1B). This is expected, as the presence of a macromolecule such as the enzyme, along with GO, enhances the monolayer’s flexibility, allowing for greater molecular adjustments under compressive forces, rather than merely increasing lateral repulsive forces. However, the addition of laccase and GO does not significantly affect the viscous-to-elastic ratio, as reflected in the phase angle comparison.

Figure 4 displays the surface potential–area isotherms for DMPA monolayers. As reported in the literature, the surface potential increases with compression due to the alignment of electric dipoles in response to the compression,^29^ reaching a maximum of 290 mV. Surface potential values can also be influenced by surface charges and the orientation of water molecules near the polar head interface.^30^ Upon the addition of laccase, and later GO, a decrease in surface potential is observed, indicating a disruption in the ability of the phospholipid chains to remain vertically ordered relative to the air–water interface. This disturbance occurs upon the adsorption of the enzyme and GO, signaling their interaction with the lipid matrix.

**Figure 4:**
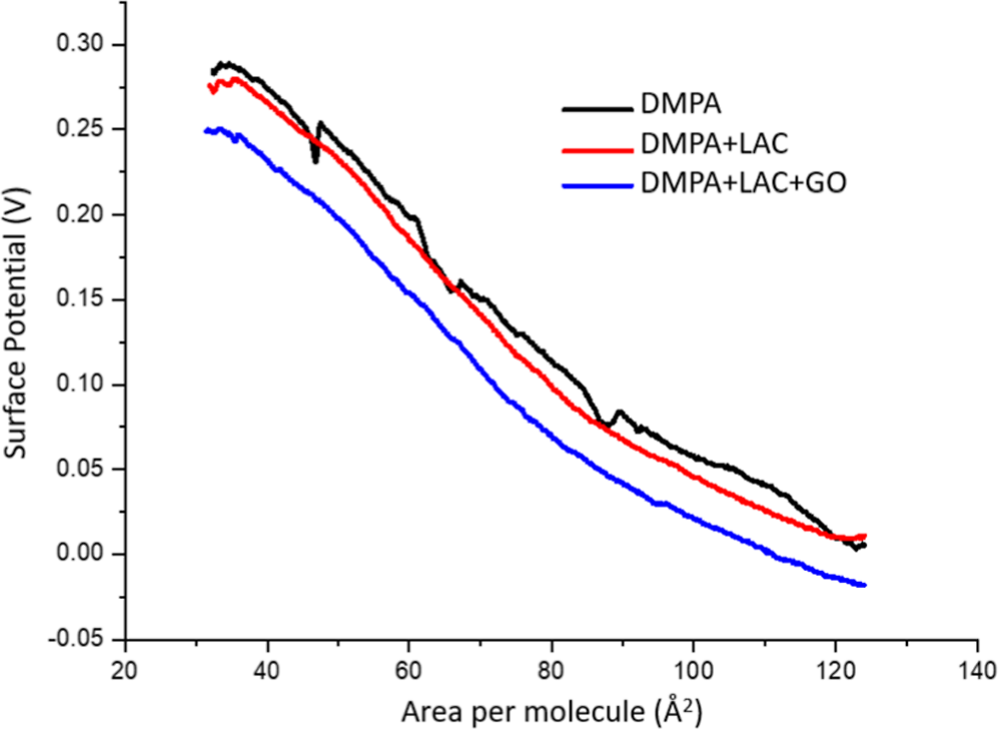
Surface potential–area isotherms for DMPA monolayers without or with laccase (0.18 μg/mL) and GO (2 μg/mL).

These results then demonstrate the presence and effects of both the enzyme and GO within the lipid monolayer. Additionally, the isotherms reveal a condensation of the monolayer, consistent with the trends observed in the surface pressure–area isotherms.

Figure 5 presents the PM-IRRAS spectra for the monolayer, divided into three panels to distinguish different wavenumber ranges. Panel 4A shows the CH_2_ stretching region, which tends to display stronger signals compared to other bands due to the technique’s selectivity. This selectivity is based on the orientation of vibrational dipoles parallel to the air–water interface.^31^ The two main bands at 2852 and 2915 cm^–1^ correspond to the symmetric and asymmetric CH_2_ stretches, respectively. The well-defined nature of these bands indicates the orientation of the CH_2_ groups. Upon the addition of laccase, slight changes are observed in the spectra. With the further addition of GO, there is a noticeable decrease in the intensity of both the asymmetric and symmetric stretches, suggesting a reduction in the all-trans conformer ratio,^32^ consistent with the surface potential data.

**Figure 5:**
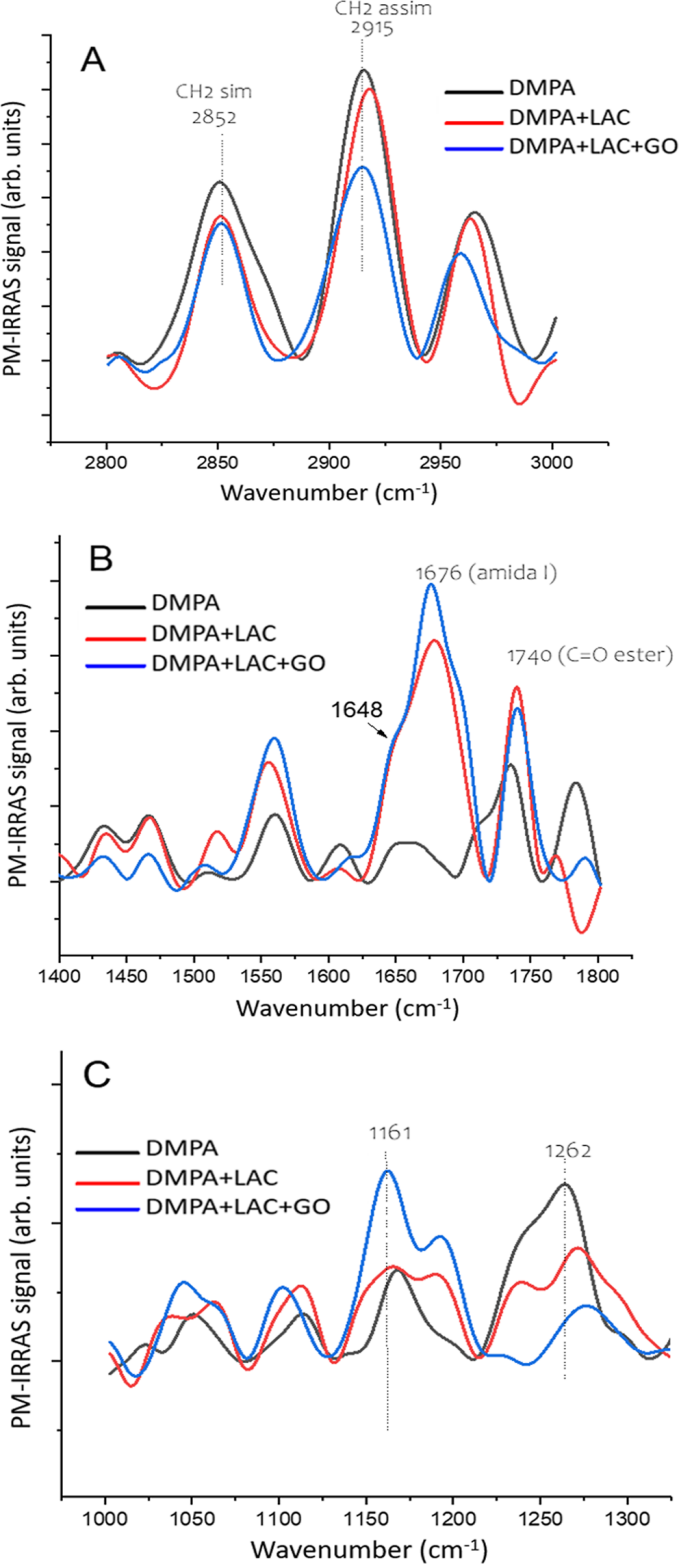
PM-IRRAS spectra for DMPA monolayers without or with laccase (0.18 μg/mL) and GO (2 μg/mL). Surface pressure = 30 mN/m. (A); 2800–300 cm^–1^ region; (B) 1400–1800 cm^–1^; (C) 1000–1300 cm^–1^ region.

Panel 4B focuses on the C=O stretching region, with a prominent band at 1740 cm^–1^ corresponding to the phospholipid ester in the polar region. When laccase is present, a broad band appears at 1676 cm^–1^, attributed to amide I bands, with a shoulder at 1641 cm^–1^ indicating the presence of α-helix structures,^33^ reflecting the secondary structure of laccase even when adsorbed onto the monolayer. The amide II band is located around 1550 cm^–1^, but it is overlapped by a pre-existing band from bare DMPA or interfacial water, resulting from differences in reflectivity between the uncovered and covered monolayer,^34^ making it less distinctive for laccase detection.

Panel 4C highlights the phosphate stretching region, typically characterized by three or four bands, with the most intense bands observed at 1161 and 1262 cm^–1^. In comparison to the other regions, this range shows the most significant changes with the presence of both laccase and GO at the interface. This finding reinforces the idea that the primary interactions between DMPA and the compounds occur at the polar headgroup/aqueous subphase interface, likely facilitated by the high concentration of ions.

The BAM images (Figure 6) reveal subtle differences when the monolayer is examined, showing small domains indicative of precollapse structures, likely due to defects formed during compression. With the addition of laccase and GO, there are no significant changes compared to pure DMPA, reinforcing the idea that these hydrophilic compounds interact primarily with the polar headgroups of the phospholipid. This interaction does not lead to significant segregation of molecules or domain formation at the interface, where cohesive forces dominate over adhesion.

**Figure 6:**
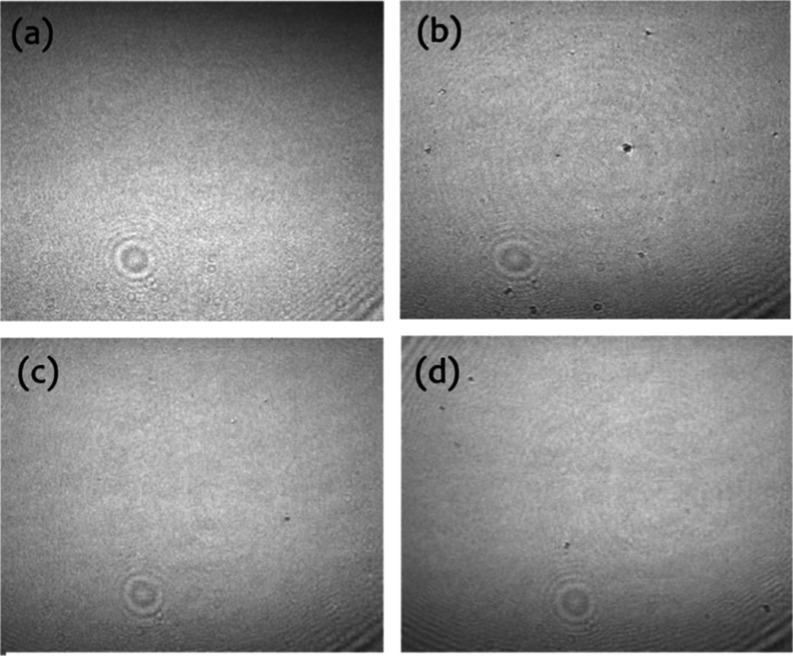
BAM images (3000 × 2400 μ m) for DMPA monolayers without (b) or with laccase (0.18 μg/mL) (c) and GO (2 μg/mL) (d). Surface pressure = 30 mN/m. Bare interface is shown for comparison (a).

As a result, the floating monolayers revealed the success incorporating of the components to the DMPA monolayer, which can be important to the properties of the supramolecular system when transferred to solid supports as LB films. The results from the Π-A isotherms and ΔV-A isotherms indicated a significant condensation of the monolayer upon the incorporation of laccase and GO, as evidenced by an increase in surface pressure at lower molecular areas. This behavior is consistent with the formation of a more compact and organized monolayer, likely due to the interactions between the enzyme, GO, and the lipid molecules. BAM images revealed homogeneous monolayers with minimal domain formation, suggesting a stable and uniform distribution of both GO and laccase within the lipid matrix.

PM-IRRAS analysis provided further insights into the molecular organization at the air–water interface, particularly the interactions between the lipid headgroups, the enzyme, and GO. The data revealed that the incorporation of GO did not significantly disrupt the secondary structure of the enzyme, indicating that the catalytic activity of laccase could be preserved within the LB films.

### 3.2 LB Films: Characterization

The first step in evaluating the feasibility of transferring LB films is to assess the tensiometric stability of the monolayer when compressed to the target transfer pressure. A surface pressure of 30 mN/m was selected for several reasons: higher surface pressures generally facilitate the transfer from the liquid to the solid interface, it is a commonly reported value in previous studies,^1^ and for bioinspired materials, this pressure corresponds to the thermodynamic conditions similar to bilayers found in cytoplasmic membranes.

Figure 7 shows the monolayers compressed to 30 mN/m. When the pressure was maintained constant, with the barriers moving back and forth, no significant barrier movement was observed, indicating the stability of the monolayer.

**Figure 7:**
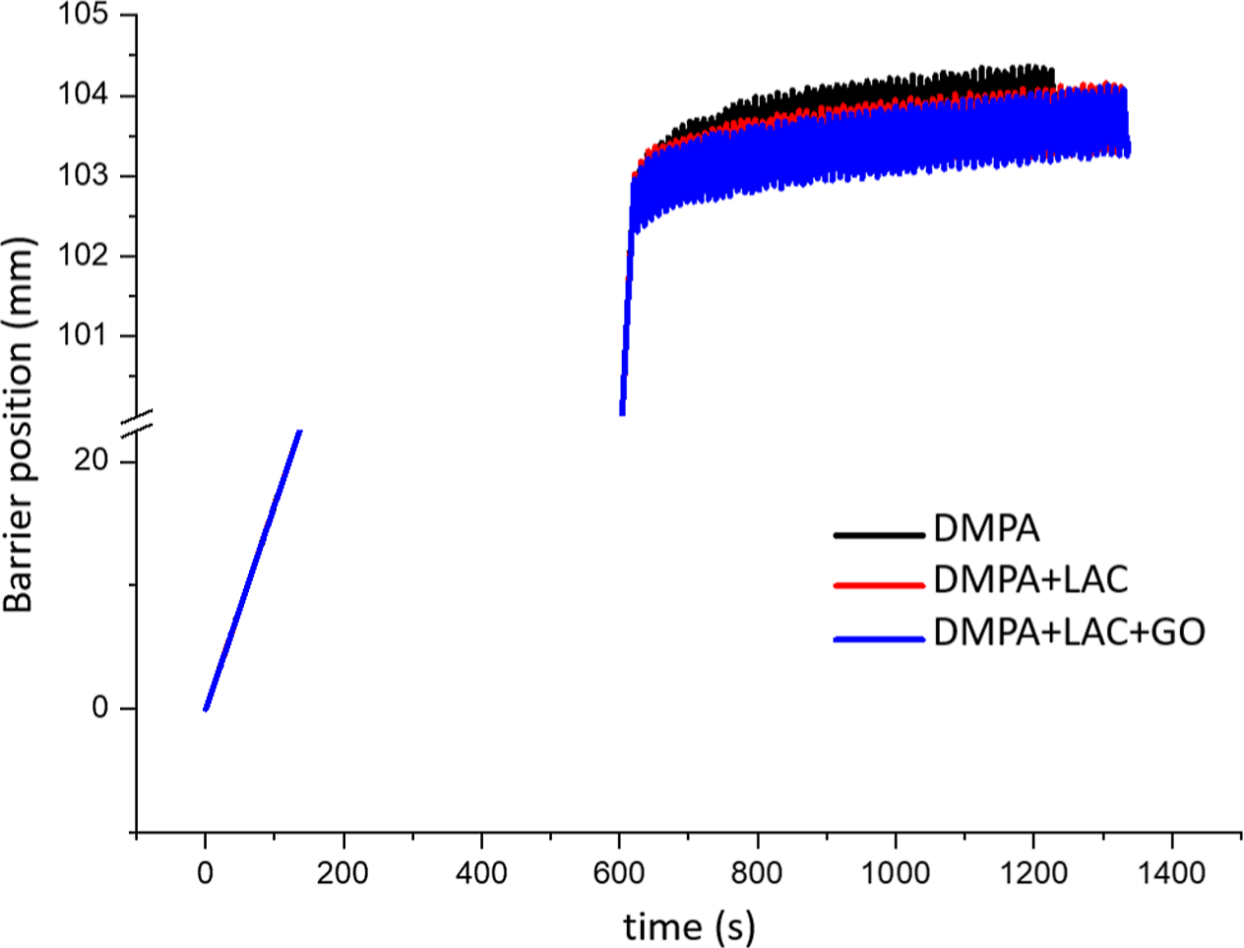
Tensiometric stability for DMPA monolayers without or with laccase (0.18 μg/mL) (c) and GO (2 μg/mL) compressed to 30 mN/m and kept with the barriers going back and forth, prior to the LB transfer.

The Table 2 presents the mass transfer from the Langmuir monolayer to solid supports measured using a quartz crystal microbalance (QCM). The data indicate the mass deposition for different film compositions, including DMPA alone, DMPA with Laccase, and DMPA with Laccase and graphene oxide, across single and multiple layer depositions.

**Table 2:** Mass Transferred from the Floating Monolayers to the LB Films Estimated with QCM

film	number of layers	deposited mass (ng)
DMPA	1	388.7
DMPA	9	4770.3
DMPA + LAC	1	1307.4
DMPA + LAC	9	2084.8
DMPA + LAC + GO	1	2208.5
DMPA + LAC + GO	9	1342.8

The selection of 9 layers for fabricating the Langmuir–Blodgett (LB) films was based on a balance between achieving sufficient film thickness for electrical and electrochemical characterization, and maintaining structural integrity and reproducibility during the transfer process. Previous studies involving LB films for functional devices have shown that multilayer assemblies (typically 5–10 layers) provide improved signal intensity, enhanced electrochemical performance, and increased surface coverage without compromising film uniformity.

Although the transfer ratio in our experiments was not exactly 1—especially in the presence of GO, where it was slightly lower due to the increased roughness and partial hydrophilicity of the GO sheets—acceptable transfer efficiency and film continuity were still achieved, as confirmed by the AFM and electrical measurements. The slight reduction in transfer ratio upon GO incorporation likely results from the heterogeneous nature of the GO-containing monolayer, which may cause partial detachment or incomplete transfer during the vertical deposition. Nonetheless, 9 layers were found to be the optimal number to produce conductive films with reproducible behavior in both the enzyme activity assay and capacitance measurements, while minimizing delamination or collapse effects observed at higher layer numbers.

The quartz crystal microbalance measurements reveal distinct trends in mass transfer efficiency for different film compositions. The deposition of nine layers of DMPA alone results in a significant mass increase, demonstrating an efficient and consistent transfer to the solid substrate. The incorporation of Laccase enhances the mass of a single-layer deposition compared to DMPA alone; however, the total mass after nine layers is lower than expected, suggesting a less effective layer-by-layer buildup. The presence of graphene oxide (GO) introduces an unusual pattern, where the mass of a single layer of DMPA + LAC + GO is substantially higher than other single-layer films, yet the nine-layer deposition results in a lower final mass than the single-layer counterpart. This indicates a poor transfer efficiency, potentially due to mass loss or desorption effects. The negative mass transfer observed in the multilayer film containing GO suggests that its incorporation disrupts the stable growth of the layers, possibly due to structural rearrangements or solubilization effects. Consequently, GO appears to hinder film stability and deposition efficiency, warranting further investigation into the molecular interactions responsible for these effects.

Figure 8 presents the PM-IRRAS spectra for LB films deposited as single layers (1 layer) and multilayers (9 layers), allowing a comparison between the two. In Panel 7A, the spectra of the LB films show bands shifted to lower wavenumbers compared to the floating monolayers, suggesting improved conformational order in the LB films. The spectra for pure DMPA LB films, which resemble the main lipid bands, are consistent with those reported in the literature.^35^

**Figure 8:**
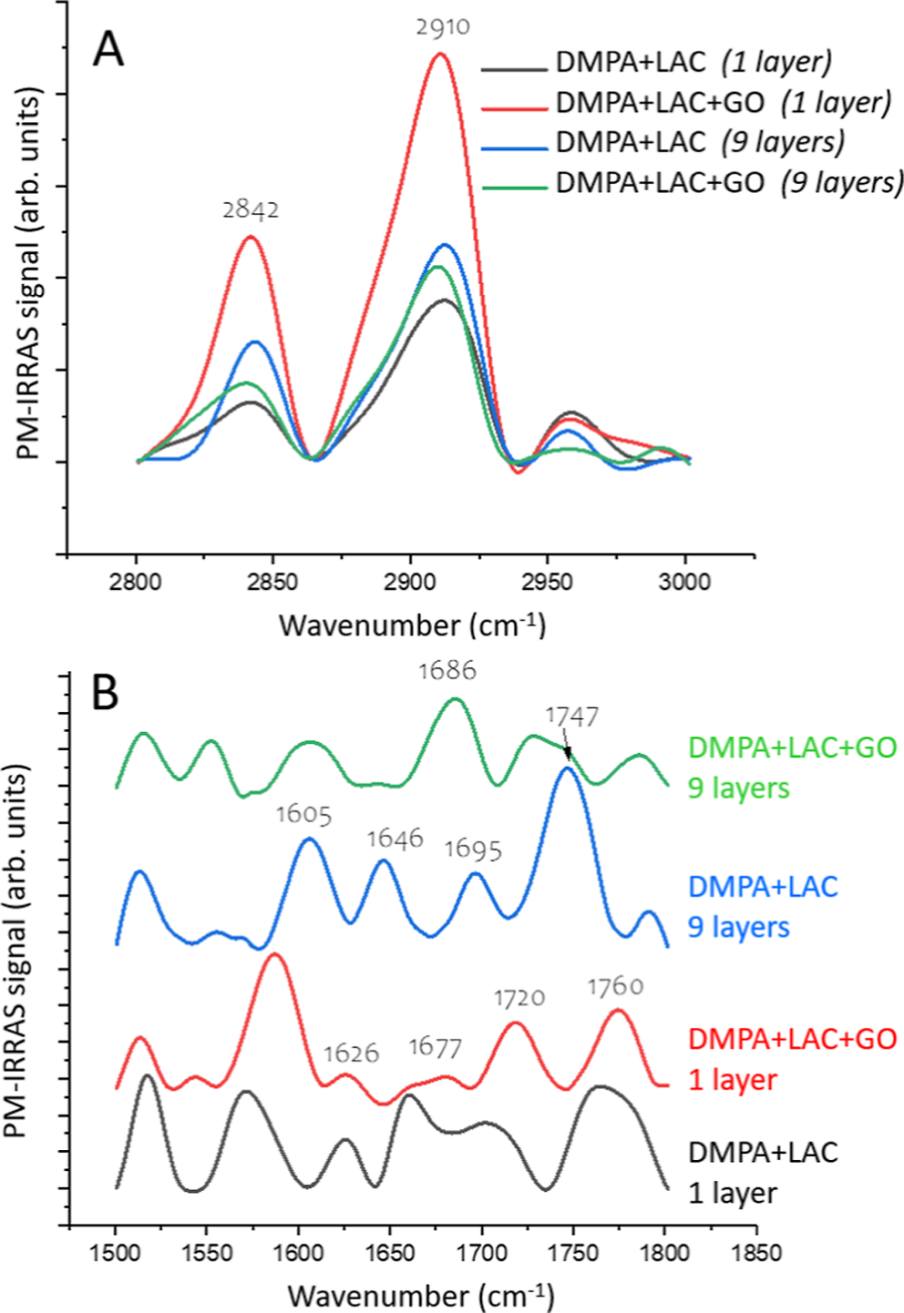
PM-IRRAS spectra for LB films transferred from floating monolayers containing DMPA with laccase (LAC) and GO, with 1 or Y-type 9-layer LB film as indicated in the caption. (A); 2800–300 cm^–1^ region; (B) 1400–1800 cm^–1^.

The shift to lower wavenumbers in the PM-IRRAS spectra for LB films compared to floating monolayers indicates improved conformational order because lower wavenumbers are associated with more tightly packed and organized molecular structures. In LB films, the molecules are transferred from the liquid interface to a solid substrate, which can promote better alignment and organization, reducing molecular disorder and enhancing the overall order of the film. This is in contrast to floating monolayers, where the molecular arrangement is typically less constrained.

In Panel 7B, significant changes are observed in the position of the amide I bands, suggesting conformational changes in the secondary structure.

For the single-layer films, the presence of a prominent band at 1626 cm^–1^ and a poorly resolved feature between 1670 and 1677 cm^–1^ is indicative of a dominant β-sheet conformation, as these wavenumber regions are commonly attributed to interstrand hydrogen bonding and antiparallel β-sheet arrangements. In contrast, the multilayer films exhibit a more complex amide I profile, with bands centered at 1605, 1646, and 1697 cm^–1^, which are more closely associated with α-helical and turn structures. Specifically, the band at 1646 cm^–1^ corresponds to the backbone C=O stretching vibration of α-helices, while the shoulder near 1697 cm^–1^ may reflect contributions from β-turns or distorted α-helices. The additional low-wavenumber feature at 1605 cm^–1^ might also be related to aggregated or unordered structures, possibly due to increased protein packing in multilayer films.

Upon incorporation of graphene oxide (GO), notable spectral changes occur: the α-helical band at 1646 cm^–1^ disappears, and the higher-frequency component at 1697 cm^–1^ shifts approximately 10 cm^–1^ toward lower wavenumbers. These shifts suggest a disruption or reorganization of the protein’s secondary structure, potentially due to interactions between GO’s oxygenated functional groups and the protein backbone or side chains. GO may influence hydrogen bonding networks, hydration layers, or steric constraints within the film, thereby destabilizing α-helical conformations and promoting partial unfolding or the formation of alternative structures such as random coils or distorted β-sheets.

Overall, the evolution of the amide I region provides insight into how protein conformation is affected by film architecture and composition. The downshift and disappearance of key bands in the presence of GO reflect changes in the local environment and protein–surface interactions, supporting the conclusion that GO incorporation alters the structural organization and possibly the functional dynamics of the immobilized enzyme.

We have also clarified that while PM-IRRAS provides valuable information on protein structure at interfaces, its spectral interpretation may be affected by molecular orientation and surface selection rules. Thus, the assignments are made with reference to known vibrational ranges but should be interpreted as indicative rather than definitive. Also, in the phosphate stretching region, the spectra were not obtained due to interference from the SiO_2_ bands of the quartz substrate.

It is important to mention that the intensity observed in PM-IRRAS spectra is indeed not an absolute measure of mass but rather an arbitrary unit based on reflectivity changes, which are influenced by multiple factors such as film thickness, surface coverage, orientation of vibrational dipoles relative to the surface, optical interference effects, and local refractive index variations. As such, PM-IRRAS is a qualitative and orientation-sensitive technique, not directly comparable in magnitude to mass-based measurements such as those from QCM.

In the case of the 9-layer film with GO, the increase in PM-IRRAS signal—despite the decrease in mass detected by QCM with successive transferences—can be attributed to optical and structural factors, such as enhanced reflectivity due to GO’s influence on surface roughness, molecular orientation, or the local dielectric environment. GO sheets can also promote favorable alignment of dipolar groups, increasing the intensity of certain vibrational modes even if the total material transferred is reduced. In fact, PM-IRRAS provides relative spectral information and should not be interpreted as a direct measure of mass or film thickness. The observed increase in intensity with GO incorporation likely reflects changes in film organization and optical properties, rather than a simple accumulation of material.

Figure 9 illustrates the determination of enzyme activity for one of the LB films. The continuous increase in absorbance at 450 nm confirms not only the presence of laccase and its successful transfer to the solid support, but also the retention of a portion of its catalytic activity. The enzyme activity was determined by selecting the linear range of absorbance increase during the first 20 min. It is important to note that the DMPA LB film without laccase showed no increase in absorbance over the first hour. Additionally, DMPA + LAC LB films transferred from monolayers on pure water also exhibited no enzyme activity, as previously discussed in this paper.

**Figure 9:**
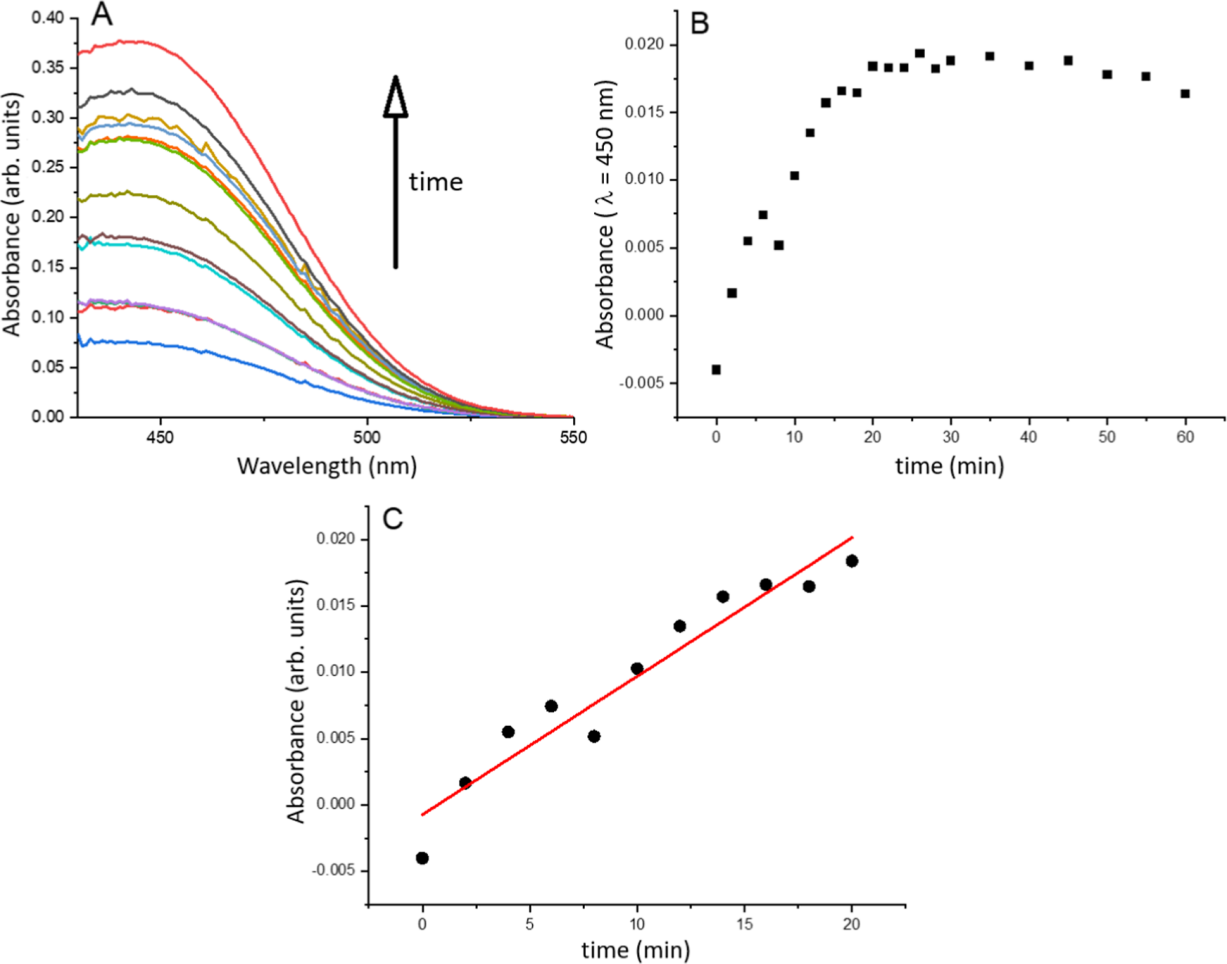
Enzyme catalytic determination for 1-layer DMPA + LAC LB film. (A) Evolution of the spectra of the system with time; (B) evolution of the absorbance at 450 nm with time; (C) plotting of the absorbance at 450 nm with time in the linear region.

Also, we have to emphasize that syringaldazine was employed in our study not as a target analyte for sensing, but rather as a well-established colorimetric probe for evaluating the retained catalytic activity of laccase after its immobilization in the Langmuir–Blodgett films. Syringaldazine is commonly used in enzyme activity assays due to its specificity for laccase, producing a distinct color change upon oxidation that can be conveniently monitored by UV–vis spectroscopy. This allowed us to confirm that the enzyme remains functionally active in the nanostructured film environment, which was the primary goal of this proof-of-concept study. Our study was not intended to develop a finished biosensor, but rather to establish a multifunctional film platform with dual electrochemical and enzymatic functionality that could be adapted in future sensor or biosupercapacitor applications.

Table 3 presents the final results of all enzyme activity measurements. As the number of layers increased from 1 to 8, enzyme activity correspondingly increased, reflecting the higher amount of enzyme transferred. This suggests that the process is not solely surface-driven, as observed in other LB systems with enzymes, where only the outermost layer is typically active.^36,37^ In this case, the inner layers also exhibit enzyme activity, and the uppermost layer does not impede the diffusion of the analyte to the enzyme’s catalytic sites.

**Table 3:** Determination of Enzyme Activity for the LB Films

composition	DMPA + LAC		DMPA + LAC + GO	
number of layers	1	9	1	9
enzyme activity Δabs_450_/min (10^–3^)	1.04	11.59	undetermined	undetermined

Interestingly, for films containing GO, whether with 1 or 9 layers, the absorbance increased rapidly within the first few seconds, and then plateaued without further increase, indicating rapid enzyme activity followed by saturation in the initial seconds. This result is the same when we obtain the activity for the free enzyme. This observation aligns with reports in the literature showing that graphene oxide can enhance the catalytic activity of confined enzymes. Since this is a diffusion-driven process, GO may facilitate the diffusion of analytes from the bulk aqueous phase to the enzymes located deeper in the film. Additionally, GO may act as a structural facilitator, as it influences the enzyme’s secondary structure. In control films containing GO but without laccase, no enzyme activity was detected, confirming that the observed activity is indeed enzyme-driven.

To contextualize the enzymatic activity observed in our system, it is worth comparing our results with literature reports involving laccase immobilization in Langmuir–Blodgett films. In a recent study by Machado et al.,^19^ laccase immobilized in ODA/P3HT LB films exhibited limits of detection (LOD) for phenolic compounds such as vanillin, catechol, and pyrogallol in the range of 0.2–2.1 μmol·L^–1^, using cyclic voltammetry. While their focus was on constructing a functional biosensor, our study emphasizes the successful preservation of enzymatic activity as a proof-of-concept for multifunctional platforms. In our system, UV–vis spectroscopic measurements showed an increase in absorbance at 450 nm corresponding to syringaldazine oxidation, with activity values ranging from 1.04 × 10^–3^ to 11.59 × 10^–3^ abs·min^–1^ for 1- and 9-layer films, respectively. Notably, LB films incorporating graphene oxide exhibited a rapid saturation profile, suggesting enhanced catalytic performance potentially driven by improved diffusion and electron transfer. These results support the viability of the DMPA + LAC + GO system for future biosensing applications, with the added advantage of electrocapacitive behavior for integrated bioelectronic devices.

To further contextualize the enzymatic activity retained in our LB films, we compared our results with those reported by Cabaj et al.,^38^ who immobilized laccase from Cerrena unicolor in Langmuir–Blodgett films composed of linoleic acid, octadecyltrimethylammonium bromide (ODTMA), and a conjugated conducting polymer. In their work, the laccase-based LB films achieved a specific activity of 328 U·cm^–2^ when using ABTS as substrate, which corresponded to approximately 30% of the activity of the native enzyme. However, when syringaldazine was used as substrate, the relative activity dropped to only 4% of that obtained with ABTS. In contrast, our LB films using DMPA and graphene oxide maintained measurable and reproducible catalytic activity toward syringaldazine, with absorbance increases ranging from 1.04 × 10^–3^ to 11.59 × 10^–3^ abs·min^–1^ depending on the number of layers. Although not intended for quantitative sensing, these results indicate a higher relative activity toward syringaldazine compared to Cabaj’s system, suggesting improved enzymatic compatibility with our film architecture. This supports the potential of our platform for future integration into biosensing applications without compromising enzyme function.

The AFM images in Figure 10 illustrate the surface morphology of different film compositions, showing variations in roughness and structural organization. The DMPA + LAC 1 Layer image likely presents a relatively smooth surface with minimal roughness, reflecting the initial deposition of laccase on the DMPA film. As the number of layers increases, DMPA + LAC 9 layers exhibits a more structured surface with increased roughness due to the accumulation of multiple layers. The incorporation of graphene oxide in DMPA + LAC + GO 1 layer introduces further domains, likely displaying dispersed heterogeneity domains within the film. As a result, the observed topographic features are consistent with increased roughness and heterogeneity induced by GO incorporation. Finally, DMPA + LAC + GO 9 layers demonstrates the most pronounced morphological changes, with increased roughness and a more interconnected network structure due to both GO incorporation and multilayer accumulation. These images collectively highlight the impact of layering and GO addition on film topography.

**Figure 10:**
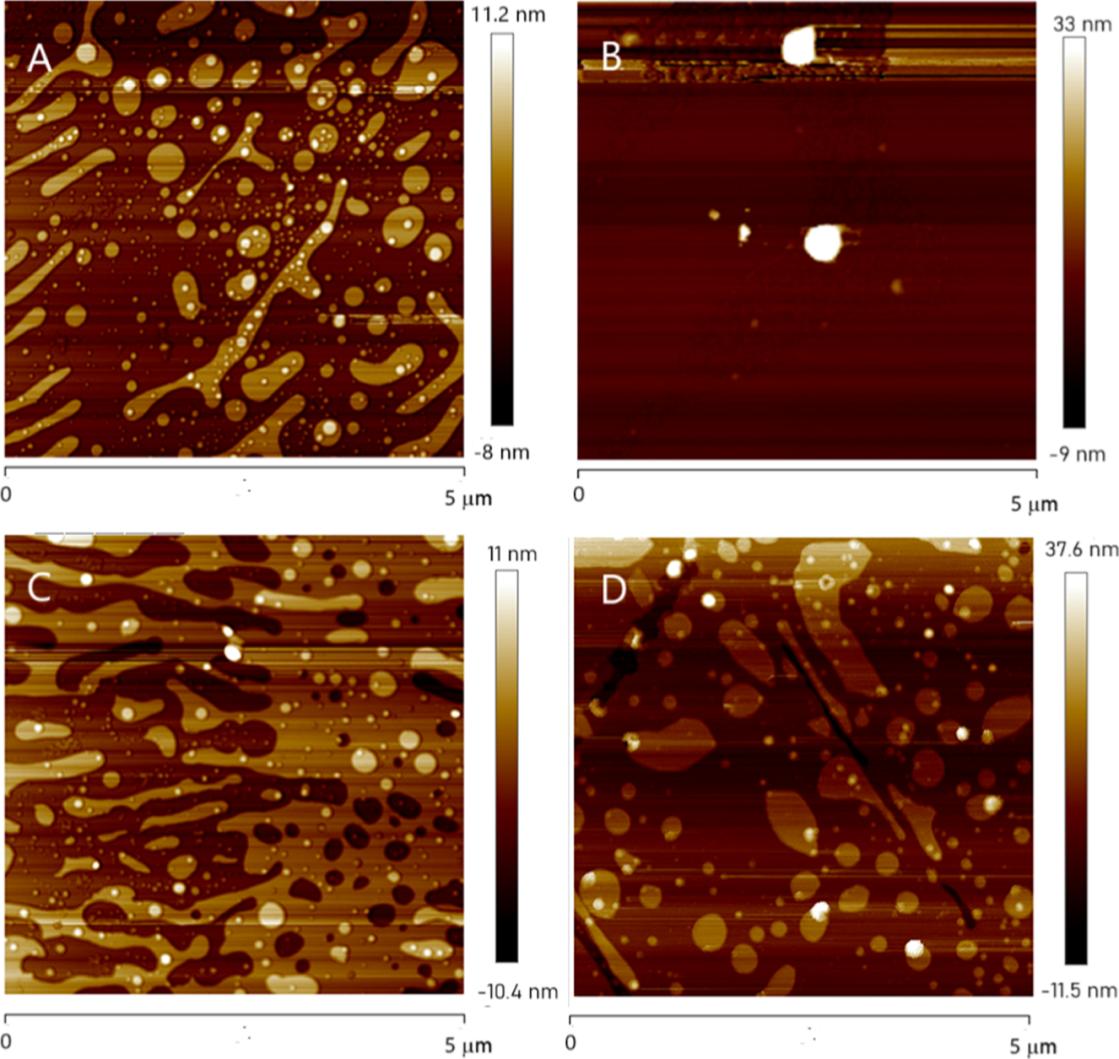
AFM images for DMPA + LAC films, with 1 (A) or 9 (B) layers and DMPA + LAC + GO, with 1 (C) or 9 (D) layers.

### 3.3 Electrochemical Properties of the Films

The electrochemical performance of the LB films was investigated to determine their suitability as electrodes for biosupercapacitors. The electrochemical measurements were performed in a three-electrode cell with 0.1 M KCl to promote ideal conditions of biocompatibility and electrochemical stability, which are essential for the development and characterization of enzyme-based biodevices. Figure 11a displays the CV curves of DMPA + LAC + GO films containing one and nine monolayers deposited on an ITO electrode, recorded within a potential range of 0–0.7 V (vs Ag/AgCl) at 50 mV/s. This potential window permits a precise evaluation of the capacitive response of the system while preserving the integrity of both the electrode and the LB film in a safe and effective range. The cyclic voltammograms exhibit a more rectangular shape, which suggests that the charge transfer process is predominantly driven by electric double-layer formation, primarily due to the contribution of graphene oxide within the film structure.^39–43^ This capacitive behavior remained consistent across different scan rates, varying from 1 to 100 mV/s (Figure 11b). Even at lower scan rates, no specific faradaic responses associated with laccase were detected, likely due to the minimal amount of enzyme present on the electrode surface.^40–43^ Additionally, a significant increase in current density was observed for the films compared to the bare ITO electrode. The presence of a single monolayer of DMPA + LAC + GO resulted in an approximately 10-fold increase in current density, while the nine-layer film exhibited a nearly 20-fold increase. These findings align with previous studies, reinforcing the role of hybrid nanostructures fabricated via the LB technique in enhancing the performance of biosupercapacitors.^13,14^ Figure 10c presents the linear correlation between current density and scan rate, suggesting efficient ion adsorption and diffusion through the film, even at higher scan rates. This behavior further confirms the capacitive energy storage mechanism of the LB-based biosupercapacitor system.^13,14^ Moreover, the observed linearity indicates uniform film deposition and strong monolayer adhesion to the ITO electrode. It is worth noting that similar CV measurements at varying scan rates were also conducted for DMPA + LAC films (data not shown), revealing an analogous voltammetric profile. Figure 10d highlights the differences between nine-monolayer DMPA + LAC + GO and DMPA + LAC LB films. The presence of GO in the film expands the CV area, yielding a higher current density compared to the DMPA + LAC film. This enhancement can be attributed to the increased surface area of the film, where GO facilitates charge transport within the monolayer by optimizing its distribution across the film structure. These results align with other reported systems, underscoring the crucial role of GO in improving electrochemical devices and advancing their overall performance.^44–47^ It is important to note that even the GO component of the LB film subjected to an electrochemical reduction step to form rGO to enhance the electrical conductivity of the hybrid system, this process did not significantly affect the enzymatic activity of Laccase immobilized on the electrode surface. This assumption is supported by the cyclic voltammetry profiles shown in Figure 10d, as the electrochemical behavior of the DMPA + LAC LB film remains consistent when compared to the DMPA + LAC + GO system, with no noticeable changes in the curve shape that would suggest enzyme deactivation. As expected, the only observed difference is an increase in current intensity after the insertion of GO in film.

**Figure 11:**
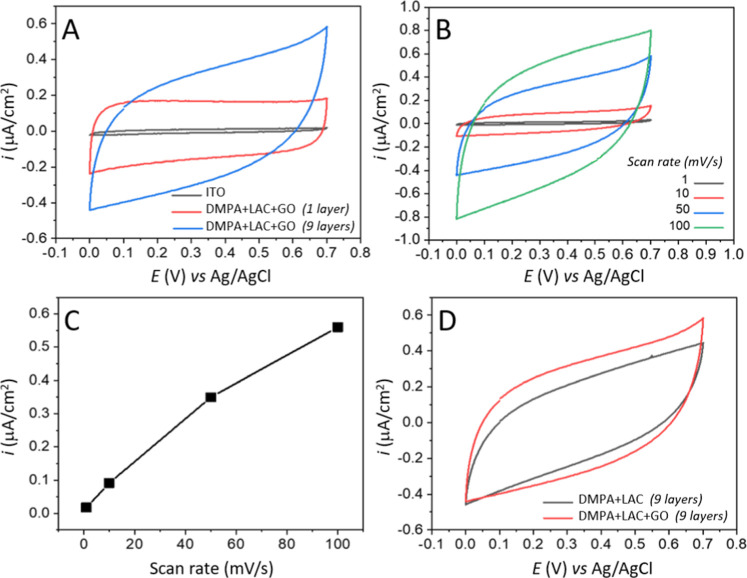
(a) Cyclic voltammograms of bare ITO and DMPA + LAC + GO LB films containing 1 and 9 monolayers at a scan rate of 50 mV/s (b) CVs recorded for a 9-layer DMPA + LAC + GO LB film at varying scan rates. (c) Linear relationship between current density and scan rates. (d) Comparative CVs of 9-layer DMPA + LAC and DMPA + LAC + GO films. All electrochemical measurements were performed in a three-electrode cell with 0.1 M KCl as the supporting electrolyte, where LB films on ITO served as the working electrode, a platinum foil as the counter electrode, and an Ag/AgCl (3 M) electrode as the reference.

Figure 12a presents the capacitance per unit area, calculated using eq 1, for DMPA + LAC + GO LB films based on CV curves obtained for films containing one and nine monolayers at different scan rates. Given the ultrathin nature of these films, capacitance values were expressed in μF/cm^2^, an appropriate unit for this type of system.^39–41^ A decrease in capacitance was observed as the scan rate increased from 1 mV/s to 100 mV/s, which can be attributed to the reduced time available for ion mobility through the film layers. This expected trend was also noted for DMPA + LAC LB films. The highest capacitance values were recorded at a scan rate of 1 mV/s, reaching approximately 16.0 μF/cm^2^ for nine-layer films and 6.0 μF/cm^2^ for single-layer films. The observed capacitance variation with different film layers highlights the impact of film organization on the electrode surface in enhancing electrocapacitive properties. These findings further validate the synergistic contribution of the DMPA + LAC + GO system, corroborating data from spectroscopic characterization and enzyme activity analysis. The electrocapacitive characteristics exhibited by these films are consistent with previous studies involving LB films of stearic acid + galactose oxidase^13^ and DMPA + GO/MnO_2_ ^14^ as biosupercapacitor electrodes, reinforcing the viability of the LB technique for developing biodevices designed for energy storage applications.

**Figure 12:**
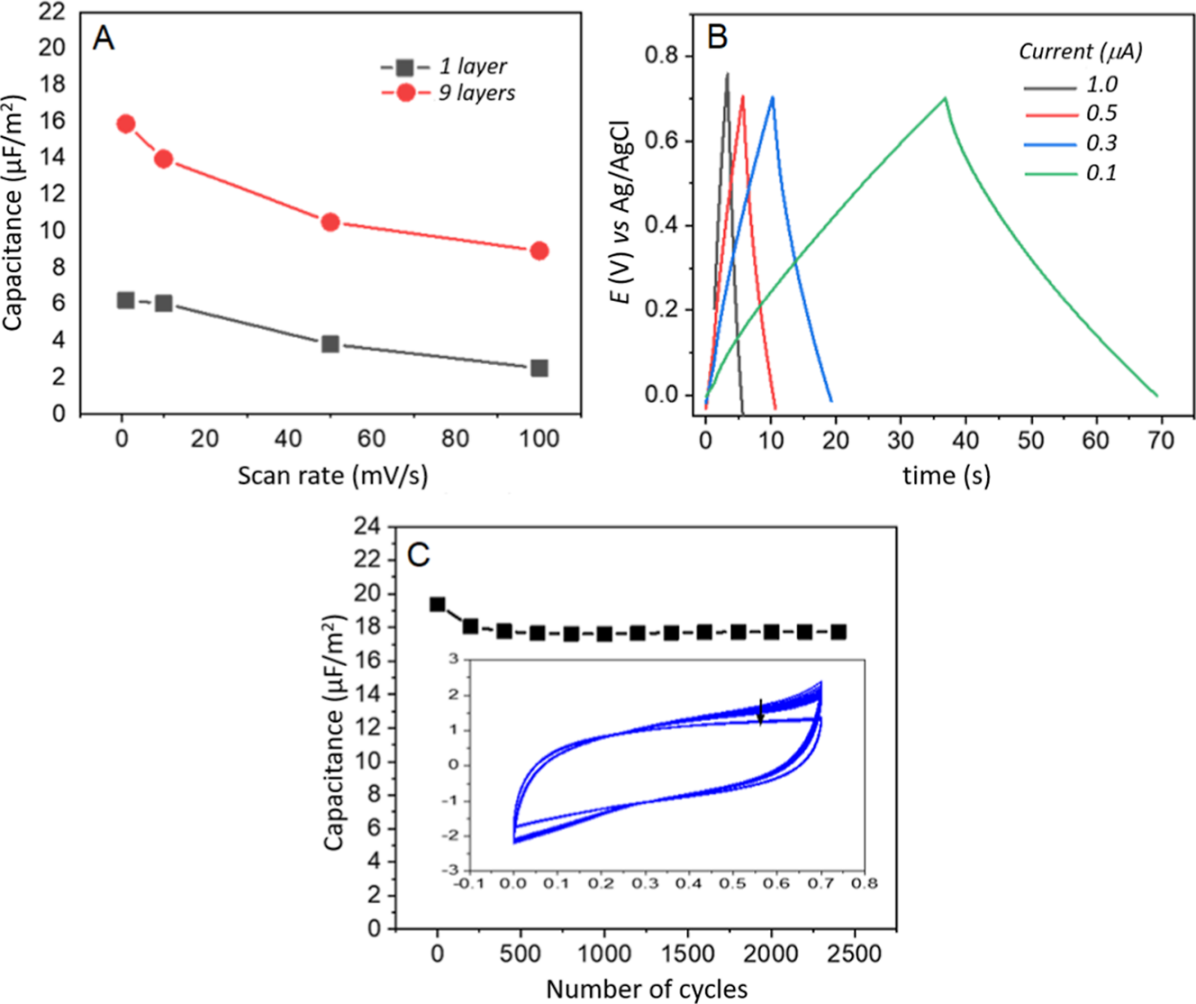
(a) Relationship between areal capacitance and scan rate for DMPA + LAC + GO LB films with 1 and 9 layers. (b) GCD curves of a 9-layer DMPA + LAC + GO LB film recorded at different constant currents, with a discharge potential window of 0.7 V. (c) Cyclic stability over 2400 continuous cycles using CV at a scan rate of 100 mV/s.

The films were also analyzed by galvanostatic charge–discharge (GCD) measurements. Figure 12b illustrates the GCD curves for a 9-layer DMPA + LAC + GO LB film at varying constant currents from 0.1 to 1.0 μA. The charge–discharge curves display a highly symmetrical triangular profile, characteristic of an electric double-layer supercapacitor. Additionally, the system demonstrated modularity in response to applied currents throughout the charge–discharge cycles, a crucial aspect for supercapacitor operation. Effective control of current flow through the electrode is essential for optimizing energy storage performance. Regarding charge–discharge duration, the maximum recorded cycle was approximately 70 s at a constant current of 0.1 μA, with a discharge potential window of 0.7 V, matching the conditions set for the CV measurements. To further assess the stability of DMPA + LAC + GO LB films, an essential factor in supercapacitor applications, continuous CV cycling tests were conducted. Figure 11c depicts the CV curves obtained after 2400 consecutive cycles at a scan rate of 100 mV/s for a 9-layer DMPA + LAC + GO LB film. Remarkably, the film retained 92% of its initial capacitance, demonstrating high electrochemical stability. The minimal capacitance loss indicates excellent capacitive retention performance, confirming the robustness of the film when deposited on an ITO electrode. Notably, the LB film remained intact, with no visible changes on the electrode surface after multiple cycles, indicating that the enzyme retained its activity within the film. This result highlights the effectiveness of the LB method in fabricating stable hybrid nanostructured films on solid substrates for use in energy storage devices.

These findings not only reinforce the potential of the LB technique for constructing hybrid electrocapacitive nanofilms but also emphasize the importance of incorporating nanomaterials into hybrid systems to develop bioinspired energy storage devices, such as biosupercapacitors. It is worth mentioning that the biosupercapacitor system developed in this study, represents an LB architecture not previously reported in the literature for energy storage applications, and therefore, there are no directly comparable systems with identical material compositions and structure that would allow for a meaningful side-by-side comparison of performance metrics such as energy and power density. Nonetheless, the specific capacitance values obtained in this study are consistent with those previously reported in our group’s earlier works,^13,14^ reinforcing the potential of enzyme-based biosupercapacitors and encouraging further exploration of hybrid bionanostructured films as innovative platforms for sustainable energy storage.

In short, the catalytic activity measurements using UV–vis spectroscopy showed that the laccase immobilized within the DMPA + GO films retained its ability to oxidize phenolic compounds. Interestingly, the enzyme’s activity was significantly enhanced in the presence of GO, likely due to the increased surface area and electron transfer capabilities provided by GO. This finding was further supported by the QCM measurements, which confirmed the stable immobilization of the enzyme (Table 3).

Additionally, vibrational spectroscopy demonstrated that the immobilized enzyme retained its structural integrity, while electrochemical analysis revealed promising potential for energy storage applications. The electrochemical data suggested that GO might contribute to preserving the enzyme’s catalytic activity over extended periods, making these films suitable for long-term use in bioelectronic devices.

The electrochemical results confirm that nanostructured films based on DMPA, laccase and GO exhibit highly promising characteristics for use as biosupercapacitor electrodes. These films demonstrated high capacitance, excellent electrochemical stability, and an efficient charge–discharge profile, reinforcing their potential for energy storage applications. The synergy between laccase and graphene oxide played a pivotal role in enhancing the capacitive properties, improving ion accessibility, and increasing charge retention. Future research should focus on exploring hybrid materials as a key strategy for advancing biobased supercapacitors. Integrating enzymatic activity with conductive polymers or metal oxides could open new possibilities for the development of high-performance, sustainable energy storage solutions.

## 4 Conclusion

In conclusion, the immobilization of laccase within DMPA + GO films was highly successful, as evidenced by UV–vis spectroscopy, QCM, and PM-IRRAS analysis. GO significantly enhanced enzyme activity by increasing surface area and facilitating electron transfer. The immobilized enzyme retained its structural integrity within the LB films, with conformational changes contributing to improved catalytic performance. Langmuir monolayer studies revealed that GO induced monolayer condensation and disrupted phospholipid chain alignment. PM-IRRAS confirmed enzyme structural integrity and identified conformational changes. Electrochemical analysis indicated the films’ potential for energy storage, with GO playing a crucial role in maintaining enzyme activity. Overall, these results demonstrate the potential of DMPA + GO composite films for biocatalysis, energy storage, and bioelectronic applications. The combination of stable enzyme immobilization, enhanced catalytic performance, and preserved structural integrity positions these films as promising candidates for long-term use in biosensors and biosupercapacitors.
